# An analysis of pain intensity, injury incidence, and their associations with socio-demographic factors in high school athletes: A cross-sectional study during the COVID-19 pandemic

**DOI:** 10.1371/journal.pone.0290587

**Published:** 2023-09-08

**Authors:** Likhit Amarttayakong, Suppakorn Ruengyangmee, Wiranya Nualkim, Pimrawee Meelam, Nattinan Rodchan, Pattama Amarttayakong, Nutcha Narom, Kittithat Sudchoo, Nitima Nulong, Arada Chaiyamoon, Sukrit Sangkhano

**Affiliations:** 1 Faculty of Education, Department of Physical Education, Khon Kaen University, Khon Kaen, Thailand; 2 School of Public Health, Walailak University, Nakhon Si Thammarat, Thailand; 3 Faculty of Medicine, Mahasarakham University, Mahasarakham, Thailand; 4 Department of Neurosurgery, School of Medicine, Tulane University, New Orleans, Louisiana, United States of America; Universiti Malaya, MALAYSIA

## Abstract

This cross-sectional study explored the relationship between pain intensity, injury incidence, and sociodemographic factors in 120 high school athletes (mean age 16.78±0.91) participating in various sports. The aims of this study are to examine the correlation between factors and Verbal Rating Scale (VRS) for pain intensity, to investigate the correlation between sport types and injured region, and to explore the correlation between injured regions and VRS during training and game competition. Using VRS, we found 64 (53.3%) participants experienced pain during training, with varying degrees of intensity. Chi-square analysis revealed a significant association between VRS scores and school grade (*p* = 0.044) and cool-down practices (*p* = 0.037). However, no significant associations were found with gender, age, or sport type. In game competition, 29 (24.2%) participants reported experiencing pain. Here, there was no significant association between VRS and any considered variables. Lower limb injuries were predominant (n = 64), particularly to the knee (n = 23) and ankle/foot (n = 18). Certain sports, like Athletics, Karate-Do, Football, and Western Boxing, demonstrated multiple injury sites, whereas Thai boxing was associated primarily with ankle and foot injuries. Pain intensity varied by injury location, with the highest ’worst pain’ reported for elbow injuries during training and knee injuries during competition. Significant associations were found between injured region and pain intensity level during both training and competition (*p*<0.001). Our findings highlight the need for injury prevention strategies and pain management among high school athletes, emphasizing proper cool-down activities. Further research is warranted to confirm these findings and explore underlying mechanisms.

## Introduction

School-based athletic programs are an essential part of many educational curriculums worldwide [1], contributing to the overall physical, mental, and social development of students [1,2]. These programs, however, carry an inherent risk of musculoskeletal injuries that can negatively impact the health and performance of young athletes [3]. Recognizing the significance of these injuries and the need for their management and prevention, a wealth of research has explored various aspects of sports injuries in student-athletes [4–7]. Understanding pain, its intensity, and related factors in the context of sports injuries in young athletes is critical, as pain is a key determinant of athletic performance and quality of life [8]. Moreover, it is an essential index of injury severity and a primary motivating factor for seeking medical attention [9]. The Verbal Rating Scale (VRS), a reliable tool for pain assessment, can facilitate accurate and effective evaluation of pain intensity experienced by student-athletes [10]. The value of the VRS lies in its simplicity, its ability to incorporate the subjective experiences of individuals, and its applicability across various contexts [11].

The COVID-19 pandemic has had significant impacts on virtually all aspects of life, including physical activity and sports practice. One of the crucial aspects that require attention is the alteration in sports practices at high school level due to the pandemic. Modifications such as reduced training intensity, restrictions on team gatherings, increased focus on individual skills, and in some instances, complete suspension of sports activities have been implemented [12,13]. These changes may have critical implications on the physical health, psychological well-being, and overall sports performance of high school athletes [14]. The limited access to regular training and competition opportunities during the pandemic could potentially exacerbate injury rates and pain intensity. Existing research suggests that abrupt changes in training load, whether an increase or decrease, can contribute to a higher risk of injury [15]. Consequently, the altered training regimens due to COVID-19 may influence the frequency, severity, and nature of sports injuries experienced by high school athletes, with associated variations in pain experiences during training and game competitive events. However, there was less well document to the analysis of pain experienced by student-athletes and its associations with sociodemographic factors, sport types, and training practices.

Therefore, the current study was undertaken with the aim of investigating pain experiences in student-athletes at an athletic school. The rationale for conducting a cross-sectional study on high school athletes during training and game competition among the COVID-19 pandemic is threefold. First, to examine the correlation between factors and VRS for pain intensity. Second, to investigate the correlation between sport types and injured region. Third, explore the correlation between injured regions and VRS. The findings could provide critical insights for designing appropriate intervention strategies to manage sports injuries and enhance the well-being of high school athletes in pandemic and post-pandemic scenarios.

## Materials and methods

### Study design and methodology

A cross-sectional design was conducted to investigate the intensity of pain experienced by high school athletes during training and game competitions. The research was conducted within the context of a high school athletics program during the COVID-19 pandemic. The study received ethical approval from the Ethics Committee of Walailak University under reference number WUEC-21-192-01, confirming adherence to established ethical guidelines and standards. In accordance with ethical guidelines, we sought informed consent from all participants before initiating data collection for our study. This entailed a comprehensive communication process wherein the details of the research project were conveyed verbally and supplemented by the dispatch of documentary correspondence. Furthermore, the study incorporated the involvement of individuals under the legal age. Consequently, to ensure adherence to established ethical norms, the requisite consent forms were transmitted digitally to the parents or guardians of these minor participants. The acknowledgment and approval were solicited through the same medium, with completed forms returned to us, thereby confirming their consent for the minors’ participation in the study.

The determination of the sample size was guided by the Krejcie and Morgan (N = 175, e = 5), and utilized a systematic random sampling approach that was proportionate to the size. In the process of participant recruitment, we successfully enrolled 120 student-athletes from a sport school. The study’s inclusion criteria required the participants to be high school student-athletes, presently studying in grades 10–12, with a prior educational experience in grades 7–9 at a sports-oriented institution. Our sample population encapsulated both genders to ensure a wide-ranging set of experiences and a comprehensive data pool. The student-athletes actively participated in at least one sport offered by the school, engaging in daily workouts for a total of 5 days per week, consisting of 2 hours in the morning and 3 hours in the evening. This could include a spectrum of sports such as football, athletics, boxing, weightlifting, sepak takraw, karate-do, Thai boxing, and wrestling, among others. Furthermore, it included student-athletes with and without the experience of at least one episode of musculoskeletal pain during training or competition within the COVID-19 pandemic timeframe. The injuries that cause pain during training or game competition include swelling, bruising, muscle deformation, injured joints, muscle strain or sprain, dislocated joints, fractured bones, fragile bones, or other conditions.

The exclusion criteria consisted of students with a history of chronic diseases or conditions, such as herniated disc, rheumatoid arthritis, diabetes, hemiplegia, stroke, and other neuromuscular disorders, which could potentially influence pain perception or injury susceptibility. Moreover, student-athletes who had undergone surgical interventions for bone or musculoskeletal injuries were also excluded. This stringent selection process ensured that the recruited participants aligned closely with the research objectives, and concurrently controlled for any potential confounding variables that could affect the study outcomes.

### Data collection

The recruitment of participants for this study was conducted from November to December of 2021, employing structured questionnaires in paper form as the primary tool for data collection. Demographic and sports participation data, including gender, age, academic year, sport type, and BMI, were collected. Moreover, a systematic record of any previous musculoskeletal injury incidents, including the corresponding pain intensity levels as experienced by the student-athletes during both training sessions and game competitions, was kept. This data was extracted from a period of one year, extending from November 2020 to October 2021. This period was notable for the COVID-19 pandemic, which significantly impacted daily life and activities in Thailand. Pain intensity was assessed using the VRS, a validated subjective measure of pain [16]. Each student rated their pain as either ’no pain’, ’mild’, ’moderate’, ’severe’, or ’worst’.

### Data analysis and statistics

Data were analyzed using descriptive statistics to summarize the demographic and sports participation data, BMI distribution, injury prevalence, and pain intensity levels. The Chi-square test was employed to investigate potential associations between the VRS and various sociodemographic factors, sport types, and warm-up and cool-down activities. Statistical significance was set at *p*<0.05. The database was managed and analyzed using IBM SPSS Statistics version 23.

## Results

### Socio-demographic data

During the study period, 120 students were attending the athletic school. There were no students who were excluded from the study due to musculoskeletal underlying diseases. A total of 120 athletic subjects were then participated, consisting of 49 (40.8%) male and 71 (59.2%) female participants, with a mean age of 16.78 ± 0.91 years (range: 15 to 18 years). The participants were categorized by academic year, with 32 (26.7%) students in grade 10, 33 (27.5%) students in grade 11, and 55 (45.8%) students in grade 12. The study population engaged in a diverse array of sports. Football had the highest number of participants (23.3%), followed by athletics (20.8%), boxing (12.5%), weightlifting (12.5%), sepak takraw (10.0%), karate-do (8.3%), Thai boxing (7.5%), and wrestling (5.0%). Body mass index (BMI) distribution among the students indicated that the majority (71.7%) had a normal BMI, while the remaining students were classified as underweight (12.5%), overweight (7.5%), obese (5.0%), and extremely obese (3.3%) (Table 1).

**Table 1:** Socio-demographic data of the participants.

Characteristics	n	%
**Sex**		
Male subjects	49	40.8
Female subjects	71	59.2
**Class year**		
Grade 10	32	26.7
Grade 11	33	27.5
Grade 12	55	45.8
**Types of sports**		
Football	28	23.3
Athletics	25	20.8
Boxing	15	12.5
Weightlifting	15	12.5
Sepak takraw	12	10.0
Karate-do	10	8.3
Thai boxing	9	7.5
Wrestling	6	5.0
**Body mass index**		
Underweight	15	12.5
Normal	86	71.7
Overweight	10	7.5
Obese	8	5.0
Extreme obese	1	3.3

We further evaluated pain intensity experienced by student-athletes during training and game competitive events. Out of the 120 student-athletes included in the sample, 40 individuals (33.3%) did not report any pain during both training and competition. However, the remaining 80 individuals (66.7%) experienced at least one episode of pain either during training or competition. Employing the VRS to assess pain intensity, 64 (53.3%) of the 120 participants experienced pain during training sessions. Within this group, students rated pain intensity as mild in 11 (17.2%), moderate in 14 (21.9%), severe in 29 (45.3%), and worst in 10 (15.6%). During competitive events, 29 (24.2%) of the participants suffered pain. Students in this group reported pain intensity as mild in 5 (17.2%), moderate in 9 (31.0%), severe in 12 (41.4%), and worst in 3 (10.4%). A Chi-square analysis was performed to investigate potential associations between the VRS and various sociodemographic factors, as well as sport types and warm-up and cool-down activities during training sessions (Table 2). The results of injury during training revealed that high school grade (*p* = 0.044), and cool-down (*p* = 0.037) were statistically associated with VRS. However, no significant associations were identified between the VRS and the other factors such as gender, age, or sport type. A Chi-square analysis which was conducted to examine the relationships between the VRS and sociodemographic factors, sport types, and warm-up and cool-down activities during competitive events indicated no significant associations between the VRS and any of these variables.

**Table 2:** Correlation between factors and Verbal Rating Scale (VRS) for pain intensity during training and game competition.

Factor as categorical variable	Injury during training (N = 64 (53.3%))					Injury during game competition (N = 29 (24.2%))				
	Verbal Rating Scale (VRS) n (%)				*p*-value (Cramer’s V)	Verbal Rating Scale (VRS) n *(%)*				*p*-value (Cramer’s V)
	Worst	Severe	Moderate	Mild		Worst	Severe	Moderate	Mild	
**Sex**										
Male	4 (15.4)	11 (42.3)	7 (26.9)	4 (8.2)	0.877	1 (11.1)	5 (55.6)	2 (22.2)	1 (11.1)	0.746
Female	6 (15.8)	18 (47.4)	7 (18.4)	7 (9.9)		2 (10.0)	7 (35.0)	7 (35.0)	4 (20.0)	
**High School Grade **										
10	1 (8.3)	4 (33.3)	4 (33.3)	3 (25.0)	0.044* (0.311)	-	-	3 (75.0)	1 (25.0)	0.726
11	1 (5.0)	14 (70.0)	1 (5.0)	4 (20.0)		1 (12.5)	4 (50.0)	1 (12.5)	2 (25.0)	
12	8 (25.0)	11 (34.4)	9 (28.1)	4 (12.5)		2 (11.8)	8 (47.1)	5 (29.4)	2 (11.8)	
**Sport types **										
Athletics	4 (25.0)	8 (50.0)	-	4 (25.0)	0.660	2 (33.3)	2 (33.3)	1 (16.7)	1 (16.7)	0.721
Karate Do	-	4 (50.0)	3 (37.5)	1 (12.5)		-	1 (33.3)	1 (33.3)	1 (33.3)	
Sepak Takraw	-	2 (33.3)	3 (50.0)	1 (16.7)		-	-	-	-	
Football	2 (11.8)	9 (52.9)	3 (17.6)	3 (17.6)		1 (30.0)	4 (40.0)	2 (20.0)	3 (30.0)	
Thai boxing	1 (50.0)	1 (50.0)	-	-		-	1 (50.0)	1 (50.0)	-	
Wrestling	1 (25.0)	1 (25.0)	2 (50.0)	-		-	2 (100.0)	-	-	
Western boxing	1 (20.0)	1 (20.0)	2 (40.0)	1 (20.0)		-	1 (25.0)	3 (75.0)	-	
Weightlifting	1 (16.7)	3 (50.0)	1 (16.7)	1 (16.7)		-	1 (50.0)	1 (50.0)	-	
**Training activities **										
Warm-up										
Never	-	-	-	-	0.095	-	-	1 (100.0)	-	0.540
More than 10 minutes	-	6 (40.0)	4 (26.7)	5 (33.3)		-	2 (28.6)	3 (42.9)	2 (28.6)	
Less than 10 minutes	10 (20.4)	23 (46.9)	10 (20.4)	6 (12.2)		3 (14.3)	10 (47.6)	5 (23.8)	3 (14.3)	
Stretching Warm-up										
Never	-	-	-	-	0.287	-	-	-	-	0.338
More than 10 minutes	2 (6.7)	14 (46.7)	8 (26.7)	6 (20.0)		-	5 (38.5)	5 (38.5)	3 (23.1)	
Less than 10 minutes	8 (23.5)	15 (44.1)	6 (17.6)	5 (14.7)		3 (18.8)	7 (43.8)	4 (25.0)	2 (12.5)	
Cool-down										
Never	-	-	-	-	0.037* (0.364)	-	-	-	-	0.074
More than 10 minutes	1 (3.1)	17 (53.1)	9 (28.1)	5 (15.6)		-	6 (35.3)	7 (41.2)	4 (23.5)	
Less than 10 minutes	9 (28.1)	12 (37.5)	5 (15.6)	6 (18.8)		3 (25.0)	6 (50.0)	2 (16.7)	1 (8.3)	
Stretching Cool-down										
Never	-	1 (100.0)	-	-	0.636	-	1 (100.0)	-	-	0.367
More than 10 minutes	3 (10.3)	12 (41.4)	9 (31.0)	5 (17.2)		-	5 (38.5)	4 (30.8)	4 (30.8)	
Less than 10 minutes	7 (20.6)	16 (47.1)	5 (14.7)	6 (17.6)		3 (20.0)	6 (40.0)	5 (33.3)	1 (6.7)	

*Significant difference (*p*<0.05).

We next examined the association between sport types and injured regions during training and game competition, and the results were shown in Table 3. Notably, lower limb injuries (n = 64) were observed to be four times as prevalent as upper limb injuries (n = 15). The highest incidence of injuries across various regions was observed in the knee (n = 23), followed by ankle /foot (n = 18), leg (n = 14), lower back (n = 11), and hip/thigh (n = 9) During training, athletics show to have 6 injuries across various regions, followed by Karate-Do, Football, and Western boxing which also exhibited 5 injuries in multiple body areas. Conversely, Thai boxing was associated with a singular injury site at the ankle and foot during training.

**Table 3:** Correlation between sport types and injured regions during training and game competition.

	Injured regions												*p*-value (Cramer’s V)
	Back		Upper limb				Lower limb				No injury n (%)	Total n (%)	
	Upper back n (%)	Lower back n (%)	Shoulder n (%)	Arm n (%)	Elbow n (%)	Wrist & hand n (%)	Hip & thigh n (%)	Knee n (%)	Leg n (%)	Ankle & foot n (%)			
**During training**													
Athletics	1 (4.0%)	2 (8.0%)	-	-	-	-	5 (20.0%)	2 (8.0%)	5 (20.0%)	1 (4.0%)	9 (36.0%)	25 (100%)	0.011* (0.330)
Karate Do	-	2 (20.0%)	1 (10.0%)	-	1 (10.0%)	-	-	3 (30.0%)	1 (10.0%)	-	2 (20.0%)	10 (100%)	
Sepak Takraw	1 (8.3%)	1 (8.3%)	-	-	-	-	-	3 (25.0%)	-	1 (8.3%)	6 (50.0%)	12 (100%)	
Football	-	2 (7.1%)	-	-	-	1 (3.6%)	3 (10.7%)	3 (10.7%)	-	8 (28.6%)	11 (39.3%)	28 (100%)	
Thai boxing	-	-	-	-	-	-	-	-	-	2 (22.2%)	7 (77.8%)	9 (100%)	
Wrestling	1 (16.7%)	-	1 (16.7%)	-	-	1 (16.7%)	-	1 (16.7%)	-	-	2 (33.3%)	6 (100%)	
Western boxing	-	-	1 (6.7%)	-	-	1 (6.7%)	1 (6.7%)	-	1 (6.7%)	1 (6.7%)	10 (66.7%)	15 (100%)	
Weightlifting	-	3 (20.0%)	-	-	1 (6.7%)	1 (6.7%)	-	1 (6.7%)	-	-	9 (60.0%)	15 (100%)	
**During game competition**													
Athletics	-	-	-	-	-	-	-	3 (12.0%)	3 (12.0%)	-	19 (76.0%)	25 (100%)	0.001* (0.321)
Karate Do	-	1 (10.0%)	-	-	-	-	-	1 (10.0%)	-	1 (10.0%)	7 (70.0%)	10 (100%)	
Sepak Takraw	-	-	-	-	-	-	-	-	-	-	12 (100.0%)	12 (100%)	
Football	-	-	-	-	-	-	-	5 (17.9%)	1 (3.6%)	4 (14.3%)	18 (64.3%)	28 (100%)	
Thai boxing	-	-	-	-	-	-	-	-	2 (22.2%)	-	7 (77.8%)	9 (100%)	
Wrestling	-	-	-	-	-	2 (33.3%)	-	-	-	-	4 (66.7%)	6 (100%)	
Western boxing	-	-	1 (6.7%)	2 (13.3%)	-	1 (6.7%)	-	-	-	-	11 (73.3%)	15 (100%)	
Weightlifting	-	-	-	-	-	-	-	1 (6.7%)	1 (6.7%)	-	13 (86.7%)	15 (100%)	

*Significant difference (*p*<0.05).

During training sessions, the highest percentage of the worst pain scale was associated with elbow injuries (50.0%), followed by shoulder and upper back injuries (33.3% each), and ankle/foot injuries (30.8%) (Table 4). In the severe pain scale, the majority of cases were found in lower back injuries (70.0%), followed by injuries of leg (57.1%), wrist/hand (50.0%) and elbow (50.0%). The highest percentage of cases with a moderate pain scale was observed in knee injuries (46.2%). During game competition, the worst pain scale was most frequently associated with knee injuries (20.0%) and leg injuries (14.3%). In terms of the severe pain scale, knee injuries accounted for the highest number of cases (n = 4), while wrist/hand injuries exhibited the highest percentage (66.7%). The highest number of moderate pain scale cases were found in knee and leg injuries, with three cases each, although the highest percentage occurred in arm injuries (100%). Significant associations were discovered between the injured regions and pain intensity levels, both during training (*p*<0.001) and game competition (*p*<0.001).

**Table 4:** Correlation between injured regions and Verbal Rating Scale (VRS) for pain assessment during training and game competition.

Regions	Verbal Rating Scale (VRS) n (%)				Total N (%)	*p*-value (Cramer’s V)
	Worst	Severe	Moderate	Mild		
**During training**	*** ***	*** ***	*** ***	*** ***	*** ***	*** ***
Shoulder	1 (33.3)	1 (33.3)	1 (33.3)	-	3 (100)	<0.001* (0.572)
Upper back	1 (33.3)	-	1 (33.3)	1 (33.3)	3 (100)	
Lower back	1 (10.0)	7 (70.0)	1 (10.0)	1 (10.0)	10 (100)	
Arm	-	-	-	-	-	
Elbow	1 (50.0)	1 (50.0)	-	-	2 (100)	
Wrist & Hand	-	2 (50.0)	1 (25.0)	1 (25.0)	4 (100)	
Hip & Thigh	1 (11.1)	4 (44.4)	2 (22.2)	2 (22.2)	9 (100)	
Knee	1 (7.7)	4 (30.8)	6 (46.2)	2 (15.4)	13 (100)	
Leg	-	4 (57.1)	1 (14.3)	2 (28.6)	7 (100)	
Ankle & Foot	4 (30.8)	6 (46.2)	1 (7.7)	2 (15.4)	13 (100)	
**During game competition**						
Shoulder	-	1 (100.0)	-	-	1 (100)	<0.001* (0.636)
Upper back	-	-	-	-	-	
Lower back	-	1 (100.0)	-	-	1 (100)	
Arm	-	-	2 (100.0)	-	2 (100)	
Elbow	-	-	-	-	-	
Wrist & Hand	-	2 (66.7)	1 (33.3)	-	3 (100)	
Hip & Thigh	-	-	-	-	-	
Knee	2 (20.0)	4 (40.0)	3 (30.0)	1 (10.0)	10 (100)	
Leg	1 (14.3)	2 (28.6)	3 (42.9)	1 (14.3)	7 (100)	
Ankle & Foot	-	2 (40.0)	-	3 (60.0)	5 (100)	

*Significant difference (*p*<0.05).

## Discussion

Socio-demographic data of the current study participants reflects an increase in diverse sports involvement among female students (Table 1), in line with current research [17]. This trend, significantly influenced by the drive for gender balance in sports, is backed by key organizations like the International Olympic Committee [18], UN Women [19], and UNESCO [20], advocating equal participation and empowerment in sports. The high participation rate in football (23.3%, Table 1) aligns with previous studies, confirming its global appeal and mass participation across diverse populations [21–23].This trend is particularly beneficial for adolescents, as previous studies have demonstrated that engagement in football can alleviate risk factors associated with overweight and obesity [24] while also enhancing various health indicators [25]. The study’s findings regarding BMI indicate that a majority of the students (71.7%) had a normal BMI, reflecting a generally healthy population. However, a portion of the students fell outside the normal range, categorized as underweight (12.5%), overweight (7.5%), obese (5.0%), and extremely obese (3.3%). Recognizing trends in adolescent athletes can guide the development of sport-specific training and nutrition programs, considering the unique hormonal changes during growth [26].

We highlighted the reported occurrence of pain among the student-athletes, with 66.7% of all participants (53.3% experiencing pain during training and 24.2% during competitive events, Table 2). Previous studies suggest that increased expectations for early sport specialization among youth athletes have led to a rise in overuse injuries, overtraining, and burnout [27–29]. It is plausible to state that coaches should be careful in planning and policy-making in youth athletic programs training. The impact of the COVID-19 pandemic on sports and athletic activities cannot be overlooked, as it has led to disruptions in training regimens, reduced physical conditioning, and altered competition schedules [30–33]. These novel hurdles can dramatically elevate stress levels in athletes [34,35], potentially culminating in psychosomatic effects that lead to modifications in muscle functionality [36]. A nationwide survey conducted on over 13,000 adolescent athletes during the COVID-19 restrictions in May 2020 reported that 40% of the participants experienced moderate to severe depression symptoms, while 37% reported moderate to severe anxiety [37]. These factors may contribute to an increased susceptibility to pain and injury [38–40] among student-athletes during pandemic. In essence, sports can act as stress inducers, given that athletes dedicate considerable time to sports-related activities, such as attending training sessions, team gatherings, travel, and competitions [41,42]. Consequently, this can instigate psychological stress in adolescents, potentially escalating muscular tension, muscular asymmetry in various body regions [43–45], muscle strength and maximal oxygen consumption [46]. It is crucial for sports medicine professionals, and coaches, to consider the potential impact of the pandemic on athlete health and well-being and implement appropriate measures to mitigate the risk of pain and injury during next challenging time.

The majority of the students indicated the severity of pain as severe or worst during training, signifying the rigorous nature of athletic preparation. Comparatively, during competitive events, the intensity distribution of pain was less skewed towards the severe end. This could potentially indicate that game-day adrenaline or focus may alleviate pain perception, or perhaps it is reflective of the acute versus chronic nature of pain experienced in these different settings [47,48]. Nonetheless, this is an area requiring further exploration to gain more profound insights. Moreover, we found that younger students in grade, presumably just beginning their high school athletic careers, were more likely to report pain during training. This finding is the same trend as a previous report [27] which found that athletes with a high degree of specialization were at a greater risk of experiencing an overuse injury compared to those with low and moderate specialization. We also observed that athletes with moderate specialization were at a higher risk of injury when compared to those with low specialization, suggesting that they might be experiencing a certain level of physical adjustment to the training demands. This presents a potential area for targeted interventions to better prepare these students for the physical demands of their sports.

The relationship between cool-down activities and pain reporting was also noteworthy (Table 2). The fact that it is statistically significant might suggest that proper cool-down procedures could influence the perception of pain or injury incidence. This underscores the importance of emphasizing adequate recovery protocols within training programs. However, it’s striking that no significant associations were found between pain intensity and other variables such as gender, age, sport type, and warm-up and cool-down activities during competitive events. This could suggest that pain perception in athletes is a complex phenomenon, potentially influenced by a myriad of factors not considered in this study. These could include individual pain thresholds, psychological factors, and precise nature and duration of training and competitive activities.

This study provides novel insight into the relationship between specific sports types and their associated regions of injury. Notably, lower limb injuries were four times as prevalent as upper limb injuries (Table 3), which is consistent with the literature on sports injuries across various disciplines [49–51]. Anatomically, the lower limbs, particularly the knee, ankle, and foot, are crucial in most sports activities as they endure significant stress and strain from bearing body weight, ensuring stability, and facilitating mobility [49]. The prevalence of injuries in these regions was likely due to the biomechanical demands placed on them during sports performance, which include running, jumping, and sudden direction changes, all of which can place significant strain on the muscles, ligaments, and joints of the lower limbs [49]. Sports such as athletics, Karate-Do, football, and Western boxing exhibited injuries across multiple body areas. This suggests a high physical demand and varied movement in these sports, which increases the risk of injuries in various body parts. For instance, in athletics, the repetitive impact and mechanical stress on the lower extremities during running and jumping activities can lead to various overuse injuries [52] On the other hand, Thai boxing demonstrated a singular injury site at the ankle and foot during training and injury at leg during game competition. This could be attributed to the sport’s unique movement patterns, which often involve intensive footwork and kicking actions, placing excessive strain on the lower extremities, particularly the ankle and foot [53]. This repetitive strain may increase the risk of musculoskeletal injuries in these regions.

A notable finding in this study is that the highest intensity of pain, as indicated by the worst pain scale, was predominantly associated with elbow injuries occurring during training sessions in Karate-Do and weightlifting (Tables 3 and 4). Additionally, shoulder injuries caused by Karate-Do, wrestling, and weightlifting, as well as upper back injuries resulting from athletic, sepak takraw, and wrestling, were closely correlated with high pain intensity. Furthermore, ankle and foot injuries were also found to be associated with significant pain (Table 4). Such elbow pain could be due to an avulsion of the triceps tendon, an injury that can occur in a wide range of sports, including weightlifting [54]. The acute pain that results from this injury may hinder the execution of a comprehensive physical examination, such as evaluating active elbow extension [55]. The exacerbation of pain intensity observed in the shoulder can be attributed to the increased vulnerability of the longhead of biceps tendon to acute rupture [56]. A further point of concern lies in the propensity of pull emphasizing larger muscle groups to cause an imbalance between the internal and external rotator cuff musculature, the rotator cuff-deltoid force couple, and the periscapular musculature. Numerous studies have suggested a link between such imbalances and an increased risk of shoulder injuries [57–59].

The severe pain scale during training sheds a different light on the distribution of pain intensity. Lower back injuries constitute the majority of cases (70.0%), trailed by leg injuries (57.1%), wrist/hand (50.0%), and elbow (50.0%) (Table 4). While elbow injuries are linked with the worst pain, they share an equal proportion of severe pain instances as wrist/hand injuries. Nevertheless, lower back and leg injuries appear to have a more substantial association with severe pain rather than the worst pain. This pattern may arise from the significant load and impact these regions endure, particularly in sports that necessitate running, jumping, or heavy lifting [60,61]. Aasa, 2017 [60], reported that lower back pain is commonly found among athletes due to the intense and repetitive stress placed on these areas, which supports our observations. Similarly, injuries to the legs, including those affecting the knees and ankles, were frequent in athletes, often resulting from overuse and high-impact activities [62]. In training, 46.2% of moderate pain cases were due to knee injuries (Table 4), often chronic from sustained wear and tear rather than severe trauma [63,64]. Common in sports involving running and jumping, these injuries arise from accumulated micro-damage in the knee joint, leading to persistent moderate pain [52,63,65]. Adjusting training load and movement mechanics can help manage these overuse injuries [15].

In the context of game competitions, the data shows a unique pattern of injury pain intensity. The worst pain reported was most frequently associated with knee injuries, followed closely by leg injuries (Table 4). This highlights the substantial physical toll of game-related activities on the lower extremities, which are often subjected to high loads and impact forces during competition [62]. In terms of moderate pain, the highest number of cases was found in knee and leg injuries, with three cases each. Notably, though, arm injuries, while less frequent, were associated with 100% occurrence of moderate pain. This suggests that while arm injuries may be less prevalent, they consistently result in a moderate level of pain when they do occur, highlighting the need for focused prevention and management strategies for this region [66].

Our study identified statistically significant correlations between injury location and pain intensity levels during both training and game competition. This result suggests that the site of an injury significantly influences the intensity of pain experienced, reaffirming findings from previous studies [67,68]. These results underscore the importance of sports medicine professionals understanding the unique risk profiles of different body parts. For instance, as our findings indicate, knee and leg injuries often result in the worst pain during games, while elbow, lower back, and leg injuries are linked to the highest pain intensities during training. Such insight can guide the design of targeted protective measures and inform appropriate training techniques for athletes [69,70]. Recognizing the specific vulnerability of certain body regions to high-intensity pain can also allow for more effective injury prevention strategies. Therefore, knee was frequently associated with exacerbated pain during athletic performance, a proactive approach may be adopted. This could entail measures such as strength training and biomechanical optimization, or the utilization of protective gear, thereby serving to safeguard the knee during activities of higher risk [63,71].

### Limitations

Our study, limited by a modest sample size of 120, does not fully represent the high school athlete population. This impacts the generalizability of our results. Injury definitions lacked distinction between acute and overuse injuries, and time off sports wasn’t recorded, which could have enhanced our understanding of the injury landscape in high school sports. The self-reported VRS for pain intensity may have introduced individual bias due to its inherent subjectivity, potentially affecting our results. The absence of a non-athlete control group restricts the depth of our understanding of the correlation between physical activity, athleticism, and pain. Key factors including growth, athleticism, and deficits in strength, flexibility, and biomechanics weren’t considered. These omitted variables, which can significantly impact injury susceptibility and recovery, represent a study limitation. Our study’s cross-sectional design precludes establishing causal relationships between variables identified and pain intensity or injury incidence. Despite these limitations, our study provides valuable insights for future, more comprehensive sports medicine research. However, caution is advised when applying our findings to broader, diverse high school athlete populations.

### Further studies

Despite these limitations, our findings offer a valuable basis for further research. Future studies with larger, diverse populations can further validate these results and identify additional contributing factors. Longitudinal studies could establish causality between various factors and the incidence of pain and injuries. Also, implementing more objective measures of pain, such as the use of validated pain scales or possibly physiological markers, would help to quantify the pain experience more accurately. Furthermore, investigating the effectiveness of specific warm-up and cool-down exercises, particularly in the context of different sports, would provide more concrete recommendations for injury prevention and pain management. Finally, exploring the psychological aspects of pain, as well as the broader impacts of COVID-19 on the overall well-being of student-athletes, would offer a more comprehensive understanding of the current state of youth sports.

## Conclusion

• Understanding Pain and Injuries: The research illuminates the prevalence and intensity of pain during training and competitive events. It also examines the relationship between specific injury locations and reported pain intensity, contributing new insights into the commonality of injuries in different sports and their associated pain levels.

• Significant Associations: The study uncovers significant associations between specific factors such as high school grade and cool-down activities with the VRS during training. It also reveals a noteworthy relationship between injured regions and pain intensity levels, both during training and competition.

• Amid the ongoing COVID-19 pandemic, this study stands as one of the few investigations of its kind. Its findings not only enhance our understanding of the experiences of young athletes during this challenging period but also provide a vital foundation for developing strategies to reduce pain and injury in student-athletes.
